# Lizard and Frog Prestin: Evolutionary Insight into Functional Changes

**DOI:** 10.1371/journal.pone.0054388

**Published:** 2013-01-16

**Authors:** Jie Tang, Jason L. Pecka, Bernd Fritzsch, Kirk W. Beisel, David Z. Z. He

**Affiliations:** 1 Department of Biomedical Sciences, Creighton University School of Medicine, Omaha, Nebraska, United States of America; 2 Department of Physiology, School of Basic Medical Sciences, Southern Medical University, Guangzhou, China; 3 Department of Biology, University of Iowa, Iowa City, Iowa, United States of America; Ecole Normale Supérieure de Lyon, France

## Abstract

The plasma membrane of mammalian cochlear outer hair cells contains prestin, a unique motor protein. Prestin is the fifth member of the solute carrier protein 26A family. Orthologs of prestin are also found in the ear of non-mammalian vertebrates such as zebrafish and chicken. However, these orthologs are electrogenic anion exchangers/transporters with no motor function. Amphibian and reptilian lineages represent phylogenic branches in the evolution of tetrapods and subsequent amniotes. Comparison of the peptide sequences and functional properties of these prestin orthologs offer new insights into prestin evolution. With the recent availability of the lizard and frog genome sequences, we examined amino acid sequence and function of lizard and frog prestins to determine how they are functionally and structurally different from prestins of mammals and other non-mammals. Somatic motility, voltage-dependent nonlinear capacitance (NLC), the two hallmarks of prestin function, and transport capability were measured in transfected human embryonic kidney cells using voltage-clamp and radioisotope techniques. We demonstrated that while the transport capability of lizard and frog prestin was compatible to that of chicken prestin, the NLC of lizard prestin was more robust than that of chicken’s and was close to that of platypus. However, unlike platypus prestin which has acquired motor capability, lizard or frog prestin did not demonstrate motor capability. Lizard and frog prestins do not possess the same 11-amino-acid motif that is likely the structural adaptation for motor function in mammals. Thus, lizard and frog prestins appear to be functionally more advanced than that of chicken prestin, although motor capability is not yet acquired.

## Introduction

Prestin, found in the membrane of mammalian cochlear outer hair cells (OHCs), is a unique voltage-dependent motor protein that does not depend on ATP and calcium [1]–[3]. Prestin confers OHCs with electromotility that is necessary for cochlear amplification [4], [5]. Amino acid sequence analyses have indentified prestin to be the fifth member of a distinct anion transporter family called solute carrier protein 26A, or SLC26A [2]. Individual members of this eleven-member family [6] serve two distinct functions. While most members are anion transporter/exchangers, prestin is the only member that functions as a molecular motor with piezoelectric capability on a microsecond time scale [3], [7]. In contrast, mammalian prestin does not appear to retain an anions transport capability [8], [9], although two recent studies suggest that prestin may be able to transport anions [10], [11]. Nevertheless, the anion transport and motor capabilities of prestin are independent [10].

Amphibian and reptilian lineages represent phylogenic branches in the evolution of tetrapods and amniotes that separated some 375 and 320 million years ago, respectively. Comparative studies suggest that the hearing organ of the amphibian and reptilian vertebrates is simple, but possesses hair cells with electrical frequency tuning capability [12], [13]. The presence of otoacoustic emissions, one of the hallmarks of the active process in the inner ear, has also been demonstrated in the ear of frog [14], [15] and lizard [16]–[19]. Although the active process in the ear of frog and lizard may be driven by a motor system in the stereocilia bundle [19], it would be interesting to determine if prestin orthologs in the inner ear of frog and lizard have acquired motor capability. Previous studies have shown that prestin orthologs from zebrafish and chicken are anion transporters and/or electrogenic divalent/chloride exchangers [20], [21] with no motor function [22]. Our recent study shows that the motor function is an innovation of mammalian prestin and the gain of this function during evolution is concurrent with diminished transport capabilities [9]. The anole lizard, *Anolis carolinensis*, is the first non-avian member of the reptile lineage to have its genome sequenced [23]. The sequenced genome fills an important gap in the coverage of amniotes, splitting the long branch between mammals and birds and allowing more vigorous analysis of amniotes evolution. We attempted to examine the motor and transport function of lizard and frog prestin with the recent availability of the sequenced genomes of frog, *Xenopus tropicalis*, and green anole lizard [23], [24]. Our goal was to determine how lizard and frog prestins are functionally and structurally different from prestins of mammals and other non-mammals. Such comparative studies may reveal molecular peculiarities underlying the mechanisms of motor and/or transport functions seen in prestin and its orthologs.

## Materials and Methods

### Cloning and Analyses of Prestin Orthologs

Identification of the genomic sequences of the *Anolis carolinensis* (*Acaro*, lizard) and *Xenopus tropicalis* (*Xtrop*, frog) SLC26A5 orthologs was done using BLAST analyses on Ensembl and NCBI genomic databases. Genomic sequence data were utilized and the resulting deduced full coding cDNAs were synthesized by GenScript USA, Inc (Piscataway, NJ). The frog- and lizard-deduced amino acid sequences of prestin were based on genomic Ensembl sequence data, which was then later verified by direct PCR-mediated genomic sequencing. If necessary site mutagenesis was performed using the QuikChange XL site-directed mutagenesis kit (Stratagene) to maintain the integrity of the exonic sequences from annotated cDNA sequences. The synthesized cDNA fragments, representing the full length coding sequences of frog and lizard, were cloned into the *XhoI* and *BamHI* sites of the expression vector pEGFP-N1 (BD Biosciences) to generate EGFP fusion-proteins. Correct orientation and reading frame were verified by sequence analyses [25]. Ortholog and paralog comparisons were done using CLUSTALW [26], Muscle and the CLC protein workbench (version 6 by CLC Bio, Cambridge, MA, USA).

### Cell Culture and Transient Transfection

Human embryonic kidney (HEK) cells were cultured in DMEM solution (Invitrogen, CarIsbad, CA), supplemented with 10% fetal bovine serum. Constructs of prestin orthologs were introduced into the dishes using lipofectamine 2000 (Invitrogen). The amount of DNA used for each 35 mm dish was 4 µg, mixed with 10 µl lipofectamine. For radioisotope uptake experiments, the cells were passaged into 24-well plates 24 hours before transfection, with cell confluence of 2×10^5^ per well. The number of cells was counted by hemacytometer (Fisher Scientific Inc., Pittsburgh, PA). The amount of DNA used for each well was 0.8 µg, added to 1.6 µl lipofectamine. Fluorescence microscopy was used to examine the membrane targeting during experiments for cell selection.

### NLC Measurements

The whole-cell voltage-clamp techniques were used to measure NLC. The experimental chamber containing the cells was placed in the stage of an inverted microscope. The cells were bathed in the extracellular solution containing (mM): 120 NaCl, 2 MgCl_2_, 2 CoCl_2_, 20 TEA, 10 HEPES, 10 4-AP. Recording pipettes, pulled from borosilicate glass with resistances between 2.5–4 MΩ were back-filled with solution containing (mM): 140 CsCl, 2 MgCl_2_, 10 EGTA, 10 HEPES. Cells with robust membrane-associated EGFP expression under fluorescence illumination were selected for NLC measurements. Membrane capacitance was measured using a two-sine-wave voltage stimulus protocol (10 mV peak at both 390.6 and 781.2 Hz) with subsequent fast Fourier transform-based admittance analysis [27] from a holding potential of 0 mV. The capacitive currents were sampled at 100 kHz and low-pass filtered at 5 kHz. Series resistance was compensated off-line. Data were acquired using jClamp (Scisoft, New Haven, CT) and analyzed with Igor (WaveMetrics, Portland, OR).

The NLC can be described as the first derivative of a two-state Boltzmann function relating nonlinear charge movement to voltage [28], [29]. The capacitance function is described as:
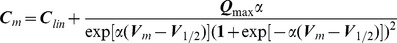
where, Q_max_ is maximum charge transfer in response to voltage stimulation, V_1/2_ is the voltage at which the maximum charge is equally distributed across the membrane, or equivalently, the peak of the voltage-dependent capacitance, C_lin_ is linear capacitance, and α = ze/kT is the slope of the voltage dependence of charge transfer where k is Boltzmann’s constant, T is absolute temperature, z is valence of charge movement, and e is electron charge.

The C_lin_, proportional to the surface area of the membrane, was subtracted and only NLC was presented as a function of voltage in the Results section. We normalized NLC by C_lin_ of the cells due to different cell sizes. Data were collected from cells whose membrane resistance was greater than 500 ΩM after rupturing. Series resistance was compensated offline. For each construct, NLC data were acquired from cells from at least three separate transfection batches.

### Motility Measurements

Voltage-evoked cell motion was measured and calibrated by a photodiode-based measurement system mounted on the inverted microscope [30]. A suction pipette (microchamber) was used to mechanically hold the cell and to deliver voltage commands [31]. Microchambers were fabricated from 1.5 mm thin-wall glass tubes (World Precision Instruments, Inc., Sarasota, FL) by a Flaming/Brown Micropipette Puller (Model P-97, Sutter Instrument Company, Novato, CA) and heat-polished to an aperture diameter of approximately 15 µm. The microchamber was mounted in an electrode holder, which was held by a Leitz 3-D micromanipulator (Leica Microsystems Inc, Bannockburn, IL). The electrical stimulus was a sinusoidal (100-Hz) voltage burst of 100 ms duration. Voltage commands of 400 mV (peak-to-peak) were used. Because the cells were approximately 50% inserted into the microchamber, the voltage drops on the extruded segment were estimated to be half of the voltage applied [32].

The magnified image of the edge of the extruded segment was projected onto a photodiode through a rectangular slit. Length changes modulated the light influx to the photodiode. The photocurrent response was calibrated to displacement units by moving the slit a fixed distance (0.5 µm). After amplification, the photocurrent signal was low-pass filtered before digitized by a 16-bit A/D board (Digidata 1322, Molecular Devices, Union City, CA). The photodiode system had a cutoff (3 dB) frequency of 1100 Hz. The sampling frequency was 5 kHz. With an averaging of 200 trials and low-pass filtering set at 200 Hz, cellular motion as low as 5 nm could be detected.

### Transport Function Assessment

Conventional radioisotope technique was used to measure transport function from HEK cells transfected with prestin orthologs from lizard and frog as described in previous studies [9], [10], [33]. Pendrin- and pEGFP-transfected cells were used as the positive and negative controls. To improve sensitivity, we measured transport function only from the cells that were positively transfected. Fluorescence-based flow cytometry was used for cell sorting. Cells whose fluorescence intensity was between 4 and 200 times greater than the none-fluorescent cells were selected. Totally 600,000 events (cells) were collected for each sample. To measure [^14^C] formate uptake, sorted cells in the 24-well culture cluster were first incubated for 30 min in the solution containing (mM): 130 NaCl, 20 HEPES, 5 KCl, 5 glucose, 2 CaCl_2_ and 1 MgCl_2_ (pH 7.3 and 305 Osm/L). Cells were then incubated in room temperature for 12 min in the solution containing (mM): 140 K-gluconate, 20 HEPES and 5 glucose (pH 7.3 and 305 Osm/L). [^14^C] formate (Moravek Biochemicals, Inc., Brea, CA) was added in this solution with a concentration of 20 µM. Cells were then washed three times with the cold K-gluconate solution without [^14^C] formate and lysed with 200 µl 0.5 M NaOH, and neutralized with 0.5 M HCl. The lysate was used for the liquid scintillation counting to determine the [^14^C] formate uptake. In each run, 3 wells were used and assayed for each plasmid. Inhibition of [^14^C] formate uptake was blocked by 1 mM of 4,4′-diisothiocyanatostilbene-2,2′-disulfonic acid (DIDS), a well-characterized blocker of anion transport [10]. The experiments were repeated in 3 separated runs. Therefore, the data in each group represented a sample size (n) of 3×3 = 9 trials for each plasmid and control.

## Results

### 1. NLC of fPres and lPres

**Figure 1:**
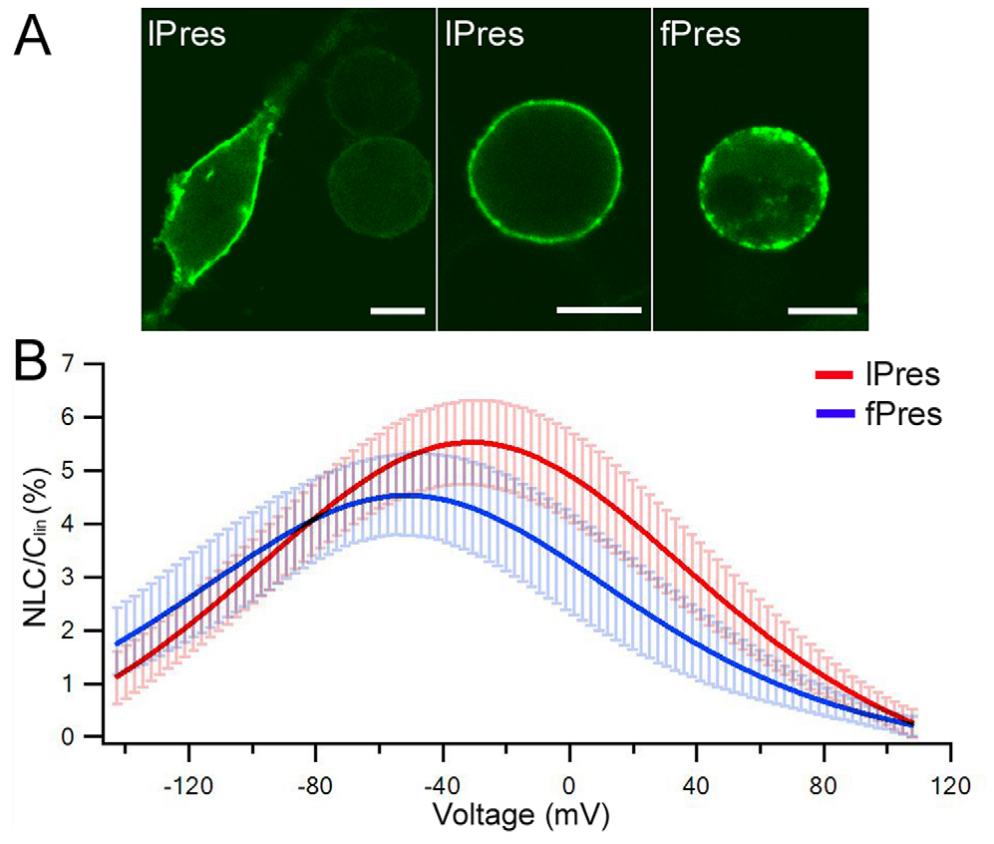
Heterogenic expression and NLC measured from prestin orthologs on transfected HEK cells. *(A)* Examples of confocal microscopy images of HEK cells transfected by lPres in the attached (left panel) and detached conditions (middle panel), and by fPres (right panel). Bar: 10 µm. *(B)* Means (heavy lines) and standard deviations (light color lines) of NLC obtained from HEK cells transfected by lPres and fPres, respectively. The mean capacitance-voltage responses were fitted with Boltzmann function. Linear capacitance (C_lin_) was used to normalize NLC.

Two essential electrophysiological properties are usually used to probe the voltage-dependent characteristics of prestin: NLC and electromotility. NLC, characterized by a bell-shaped dependence on membrane potential with a peak between −70 and −20 mV [28], [29], reflects voltage-dependent charge movement arisen from the redistribution of charged “voltage sensors” across the membrane. Electromotility is the result of the “actuator” that undergoes a conformational change after the voltage sensor detects transmembrane potential changes [3], [5]. We examined these two electrophysiological properties from HEK cells transfected by lPres and fPres with EGFP-tagging. Membrane expression of lPres and fPres was examined using confocal microscopy. Both lPres and fPres exhibited robust membrane expression 24 hours after transfection. Some examples of membrane expression of HEK cells transfected by lPres and fPres respectively are presented in Figure 1A. NLC was recorded from EGFP-positive transfected HEK cells and the magnitude of NLC was normalized by C_lin_ to minimize the influence of different cell sizes. Figure 1B (in red) shows means and standard deviations of NLC response recorded from ten lPres-transfected HEK cells. As shown, lPres showed a robust bell-shaped dependence on membrane potential with the peak capacitance near −40 mV. We obtained four parameters (Q_max_, C_lin_, V_1/2_ and *z*) from nonlinear curve-fitting of the NLC response using the first derivative of the Boltzmann function. These four parameters are often used to define the magnitude and voltage-dependency of NLC [3], [5]. As in all previous studies [8]–[11], we used C_lin_ to normalize Q_max_ due to different cell sizes. The yielded mean values and standard deviations of the parameters are: Q_max_/C_lin_ = 8.1±2.2 fC/pF, *V*_1/2_ = −31.4±9.9 mV, and *z* = 0.5±0.06. We also obtained NLC measurements from fPres. Figure 1B presents means and standard deviations of NLC response recorded from eight fPres-transfected HEK cells. Parameters derived from the curve fitting with Boltzmann function are: Q_max_/C_lin_ = 4.9±1.1 fC/pF, *V*_1/2_ = −52.2±7.2 mV, and *z* = 0.58±0.18.

**Figure 2:**
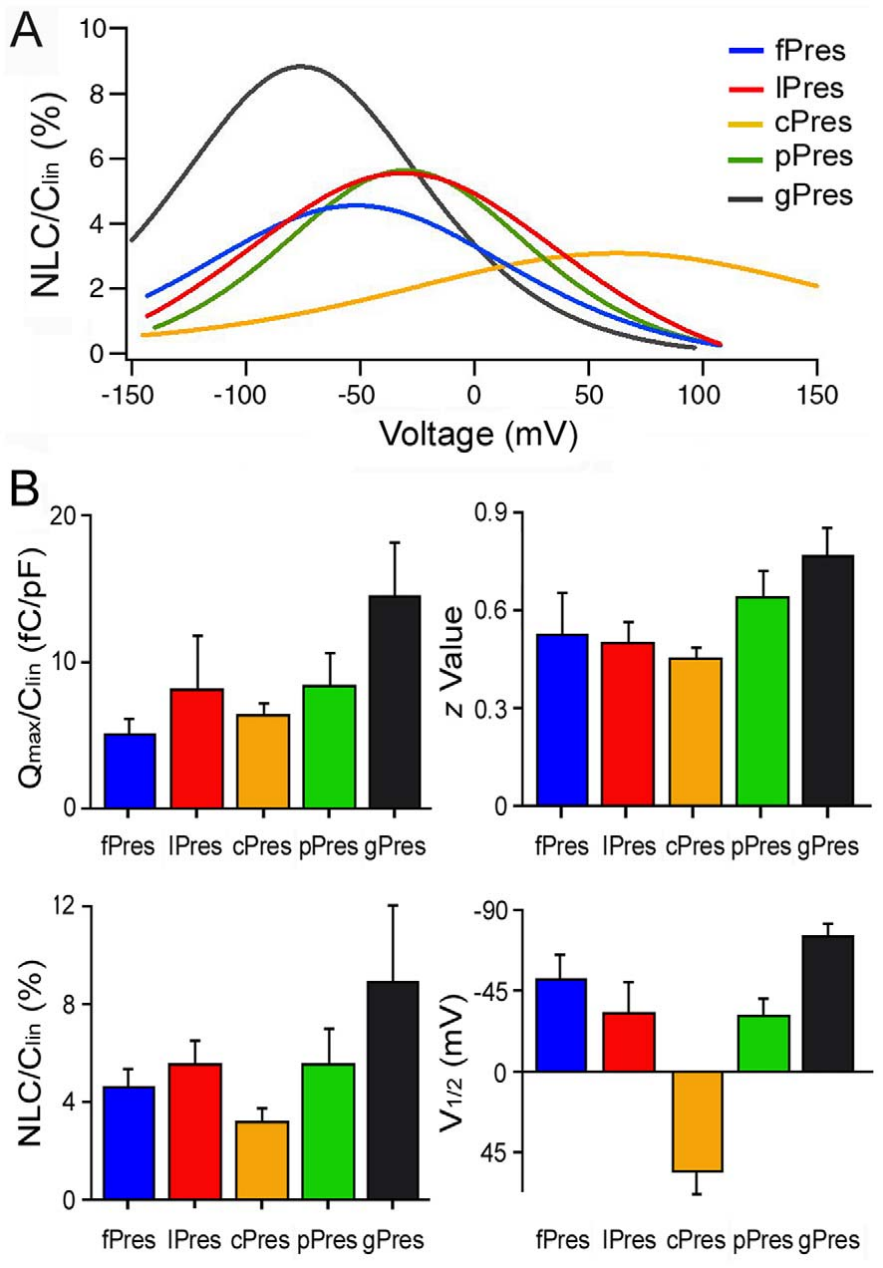
Voltage-dependent NLC of prestin and its orthologs. (*A*) Means of NLC responses measured from gPres (n = 11), pPres (n = 9), cPres (n = 12), lPres (n = 10) and fPres (n = 8). The mean capacitance-voltage responses were fitted with Boltzmann function. (*B*) Four parameters obtained from curving fitting using Boltzmann function.

We compared voltage-dependent properties of gerbil prestin with prestin orthologs from platypus, chicken, lizard and frog. Figure 2A shows the means of NLC responses recorded from HEK cells transfected by prestin and its orthologs. Parameters derived from the curve fitting with Boltzmann function are summarized in Figure 2B. It is apparent that V_1/2_ of lPres and fPres was significantly shifted toward the negative potentials with reference to chicken prestin (cPres). Many studies have shown that V_1/2_ can be altered by different factors that influence the anion-binding capability of prestin [5]. Thus, the shift of V_1/2_ toward negative potentials may suggest an improvement in anion-binding capability of lPres and fPres with reference to cPres. NLC/C_lin_ and Q_max_/C_lin_ are both related with charge density or the amount of voltage sensors available. The larger the values, the more charges are translocated during voltage stimulation. As shown in Figure 2B, both NLC/C_lin_ and Q_max_/C_lin_ of lPres were significantly larger than those of cPres and were similar to those of platypus prestin (pPres). The z value is associated with the slope of the capacitance-voltage function. The steeper the curve (function), the larger the z value. Like V_1/2_, the z value is an important criterion to assess prestin’s voltage-sensing ability [5]. In a typical outer hair cell or gPres-transfected HEK cell, the z value is ∼0.8. This number represents a single elementary charge moved across the 0.8 of the membrane electric field (or two charges moved across the 0.4 of the membrane electric field). As seen in Figure 2B, the mean of z value of lPres and fPres was 0.5 and 0.58, respectively, both being greater than that of cPres. Taken together, it appears that lPres and fPres are evolutionarily more advanced than cPres.

### 2. Voltage-dependent Somatic Motility of fPres and lPres

**Figure 3:**
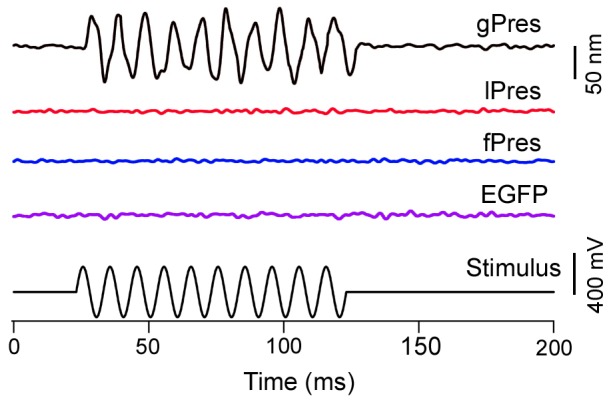
Examples of motile responses measured from HEK cells transfected by gPres, lPres, fPres, and EGFP vector, respectively. The cell was approximately 50% inserted into the microchamber and length change of the extruded segment was measured by a photodiode-based displacement-measurement system. The electrical stimulus (bottom panel) was a 100-Hz sinusoidal voltage burst with duration of 100 ms. No time registered motile response was seen from lPres-, fPres- and EGFP-transfected HEK cells. Motility responses measured were observed from gPres-transfected HEK cells. The responses were the results of 200 averages.

The motor function is mediated by the “actuator” in the molecule and manifested as length changes in OHCs or dimensional alternations in prestin-transfected cells. We measured electromotility of the transfected HEK cells using the microchamber technique [1], [9], [31]. The estimated peak-to-peak voltage dropped on the extruded segment was ±200 mV [32]. This voltage was sufficient to evoke a saturated response even if the voltage dependency of NLC response was significantly shifted toward the positive voltage (e.g., cPres). Ten HEK cells transfected with gPres (gerbil prestin) were used as a positive control for measuring motility. As shown in Figure 3, HEK cells transfected with gPres exhibited large cycle-by-cycle response to the sinusoidal voltage stimulation. Ten lPres-transfected cells, all exhibiting clear membrane labeling, were tested for electromotility using sinusoidal voltage burst. No time-registered and stimulus-following responses were detected in any of the lPres-transfected cells measured with the system resolution of 5 nm. An example of lack of motile response is presented in Figure 3. We also measured motility from ten fPres-transfected cells using the same voltage protocol. None of the cells measured showed any time-registered response to the sinusoidal voltage stimulus (Fig. 3).

#### Transport function of fPres and lPres

Radioisotope technique using [^14^C] formate as the substrate was used to determine whether lPres and fPres prestin was able to transport formate across the membrane. Eight constructs including human pendrin, zPres (zebrafish prestin), fPres, lPres, cPres, pPres, gPres and EGFP-N1 were tested. The human pendrin (SLC26A4), a paralog of prestin and a known anion transporter [34], was used as positive control, while an EGFP-N1 expression plasmid was used as negative control.

**Figure 4:**
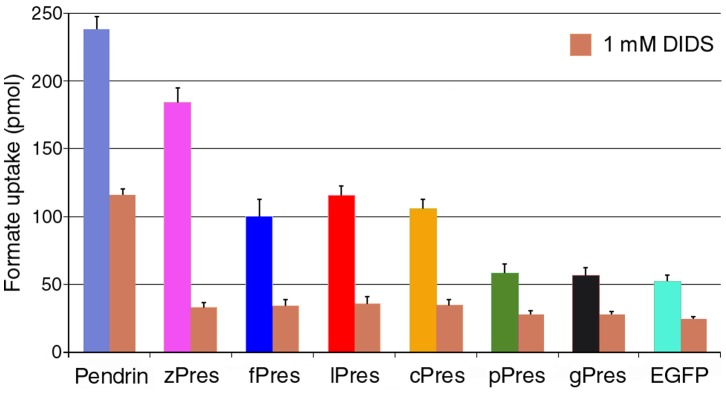
Transport activity measured using radioisotope technique with [^14^C] formate as the substrate. Human pendrin was used as a positive control and EGFP plasmid was used as a negative control. Inhibition of formate uptakes by DIDS was also presented. All the data were acquired from 3 wells in each plate and repeated 3 times.

HEK cells transfected with pendrin showed a robust increase in format uptake compared to the background formate uptake in the EGFP-N1 transfected cells (Fig. 4). Formate uptakes of the HEK cells transfected with lPres and fPres were also significantly higher than the background (one-way ANOVA, P<0.01). However, the formate transport capability of lPres and fPres was significantly less than that of zPres and was comparable to that of cPres. As we demonstrated before [9], cells transfected with pPres and gPres did not show a significant increase in formate uptake above the control value. We also measured transport activity of all the constructs in the presence of DIDS, an anion transporter blocker. As shown in Figure 4, formate uptakes of all the constructs were significantly reduced by DIDS (p<0.01 in all groups). Although the background formate uptake by the cells transfected by EGFP-N1 was also reduced, significant reduction of transport function in the presence of DIDS suggested that formate uptake was mediated by transporters.

### 3. Amino acid Sequence Difference Among Mammalian Prestin, fPres and lPres

**Figure 5:**
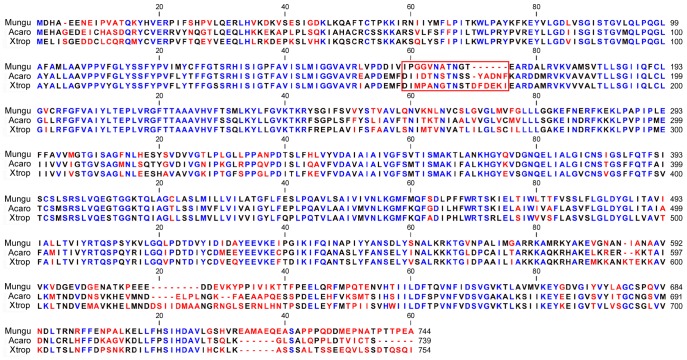
Alignment of consensus amino acid sequences of SLC26A5 of gerbil (Meriones unguiculatus, Mungu), frog (Xenopus tropicalis, Xtrop) and anole lizard (Anolis carolinensis, Acaro). Color of each residue represents identity at the residue among three different species. Blue: Full identity at a residue; Black: Partial identity (2/3 sequences) at a residue; Red: complete disparity at a residue. Gaps in the aligned sequences are indicated by the dashed line. The red box marks the motif that is remarkably conserved among mammalian species but highly variable among non-mammalian orthologs. This area may reflect a structural adaptation that facilitates gain of motor function in mammalian prestin.

We analyzed the amino acid sequence differences among gPres, lPres and fPres to understand how they are structurally and functionally different and how their prestins evolve. Figure 5 shows the consensus amino acid sequences of gPres, fPres and lPres. As shown, gPres has 744 amino acids, whereas fPres and lPres have 754 and 739 amino acids, respectively. Comparison of the deduced sequences from fPres and lPres was made with the prestin peptide sequences of gerbil, platypus (747 residues), chicken (742 residues), and zebrafish (*Danio rerio*, *Dreri*) (739). As summarized in Table 1, lPres shares 60% homology to gPres while the fPres’ homology to gPres is 57%. The amino acid identity between fPres and lPres is 70%. Homology among fPres, lPres, cPres and zPres is between 60% and 70%. Comparisons were also made among these three sequences for the amino cytoplasmic tail (residues1–65), the SulPtp domain (the hydrophobic core – residues 66–515) and the carboxyl cytoplasmic tail (residues 516–744) (Table 2). The SulPtp domain has the greatest homology with identity being approximately 70–80% with the fPres and lPres having a positivity of 89%. Both the amino and carboxyl cytoplasmic tails exhibit 33–50% and 43–60% identity, with the gPres having the lowest homology with the other two orthologs (Table 2).

**Table 1:** Amino acid sequence comparisons of gerbil, platypus, lizard, frog, chicken and zebrafish prestin orthologs.

	Gerbil			Platypus			Lizard			Frog			Chicken			Zebrafish		
	Id	Pos	Gap	Id	Pos	Gap	Id	Pos	Gap	Id	Pos	Gap	Id	Pos	Gap	Id	Pos	Gap
Gerbil				578/747	646/747	10/747	441/741	567/741	18/741	422/737	555/737	19/737	386/743	499/743	17/743	390/728	510/728	21/728
(744 residues)				77%	86%	1%	60%	77%	2%	57%	75%	3%	52%	67%	2%	54%	70%	3%
Platypus	568/725	631/725	10/725				451/722	570/722	13/722	431/732	565/732	21/732	370/708	485/708	15/708	393/735	512/735	23/735
(747 residues)	78%	87%	1%				62%	79%	2%	59%	77%	3%	52%	69%	2%	53%	70%	3%
Lizard	441/741	567/741	18/741	456/742	577/742	19/742				513/733	614/733	10/733	427/721	540/721	8/721	448/732	549/732	22/732
(739 residues)	60%	77%	2%	61%	78%	3%				70%	84%	1%	59%	75%	1%	61%	75%	(3%)
Frog	422/737	555/737	19/737	434/747	571/747	24/747	513/733	614/733	10/733				440/731	555/731	10/731	444/723	557/723	18/723
(754 residues)	57%	75%	3%	58%	76%	3%	70%	84%	1%				60%	76%	1%	61%	77%	(2%)
Chicken	386/743	499/743	17/743	370/708	485/708	15/708	423/710	536/710	7/710	440/731	555/731	10/731				496/719	582/719	16/719
(766 residues)	52%	67%	2%	52%	69%	2%	60%	75%	1%	60%	76%	1%				69%	81%	(2%)
Zebrafish	390/728	510/728	21/728	393/735	512/735	23/735	448/732	549/732	22/732	444/723	557/723	18/723	499/732	586/732	16/732			
(739 residues)	54%	70%	3%	53%	70%	3%	61%	75%	3%	61%	77%	2%	68%	80%	2%			

Id = identities; Pos = Positives.

**Table 2:** Amino acid sequence comparisons of the three structural features of the gerbil, lizard and frog prestins.

PeptideDomain	Species(peptide length)	Gerbil			Lizard			Frog		
		Id	Pos	Gap	Id	Pos	Gap	Id	Pos	Gap
AminoCytoplasmicTail	Gerbil				26/66	42/66	1/66	22/66	40/66	1/66
	(65 residues)				(39%)	(64%)	(2%)	(33%)	(61%)	(2%)
	Lizard	26/66	42/66	1/66				33/66	44/66	1/66
	(65 residues)	(39%)	(64%)	(2%)				(50%)	(67%)	(2%)
	Frog	22/66	40/66	1/66	33/66	44/66	1/66			
	(65 residues)	(33%)	(61%)	(2%)	(50%)	(67%)	(2%)			
SulPtpDomain	Gerbil				310/448	369/448	5/448	302/449	368/449	6/449
	(450 residues)				(69%)	(82%)	(1%)	(67%)	(82%)	(1%)
	Lizard	310/455	369/455	12/455				349/449	398/449	1/449
	(455 residues)	(68%)	(82%)	(3%)				(78%)	(89%)	(0%)
	Frog	308/449	368/455	6/455	349/449	398/449	1/449			
	(455 residues)	(67%)	(82%)	(1%)	(78%)	(89%)	(0%)			
CarboxylCytoplasmicTail	Gerbil				105/227	156/227	12/227	99/228	149/228	12/228
	(229 residues)				(46%)	(69%)	(5%)	(43%)	(89%)	(6%)
	Lizard	102/227	152/227	12/227				133/221	174/221	8/221
	(227 residues)	(46%)	(69%)	(5%)				(60%)	(79%)	(4%)
	Frog	99/228	149/228	13/228	133/221	174/221	8/221			
	(234 residues)	(43%)	(65%)	(6%)	(60%)	(79%)	(4%)			

Id = identities; Pos = Positives.

Interestingly, the mammalian sequences share the greatest homology (>95%) with variations primarily restricted to the amino (residues 1–65) and carboxy (residues 516–744) termini. The carboxyl-end of these peptides exhibits the greatest variation among species. A relatively high amino acid sequence homology is also shared by other mammalian species, the prototherian platypus and the metatherian opossum species, with the mammalian prestin proteins, as previously reported by us and others [35]–[39].

## Discussion

It is suggested that the evolution of mammalian prestin motor function occurred as a result of multiple episodic adaptive events [40]. The initial phylogenetic analyses of vertebrate prestin genes [35]–[37] was further refined by Liu et al. [40] who revealed that the inceptive episodic adaptive event occurred with the emergence of tetrapods. The acquisition of motility properties being obtained in platypus and opossum [9], [40] with the SulPtp region heterogeneity became relatively fixed in the eutherians [35]–[37], [40]. Our investigation focused on the functional properties of lizard and frog prestins to determine how lizard and frog prestins are functionally and structurally different from prestins of mammals and other non-mammalian vertebrates. We evaluated the characteristics of lPres and fPres for anion transport, NLC and motor capability. We showed that both lPres and fPres had robust NLC, whose magnitude was greater than that of chicken prestin and similar to that of platypus. The peak of NLC (V_1/2_) of fPres and lPres were also similar to that of mammalian prestins. If functional evolution of prestin is characterized by a graduate gain of NLC and a shift of voltage dependency from positive to negative voltages as we demonstrated in a previous study [9], then fPres and lPres are evolutionarily more advanced than that of chicken prestin. Interestingly, despite of their relative functional advancement in evolution and their similarity to pPres in their NLC characteristics, neither lPres nor fPres demonstrated motor capability. Their lack of motor-like activity again shows that the motor function of prestin is a newly derived molecular property exclusive to mammals.

Frog or lizard prestin has retained the presumably ancestral capability to transport anions. Their transport activity was not significantly different from that of cPres. In the previous study we showed that there was a reciprocal trend between NLC and anion transport properties during functional evolution of prestin [9]. Inverse changes are observed such that as transport function diminishes from zebrafish to gerbil, NLC becomes more prominent along with a characteristic shift of V_1/2_ from positive to negative voltages. The fact that fPres and lPres can transport anions suggests that it is most likely that all non-mammalian prestin orthologs serve as transporter. It may also suggest that acquisition of motor capability during evolution has to be concurrent with the loss of anion transport capability, despite of the fact that motor and transporter functions are independent and have different structural bases [9], [10], [39].

NLC is generated by charge movements, similar to the gating current seen in voltage-dependent ion channels. Intracellular anions such as Cl^-^ and HCO_3_^−^ may act as the voltage sensor of prestin [8] or work for allosteric modulation [41]. In either model, the translocation of charges triggers conformational changes of the prestin molecule that subsequently change its surface area in the plane of the plasma membrane. The presence of a NLC in fPres, lPres and other non-mammalian prestin orthologs suggests a common mechanism of charge movement for both mammalian prestin and its non-mammalian orthologs. We should note that voltage-dependent charge movement, or gating current, is not unique to prestin. Voltage-dependent ion channels and some transporters also possess gating currents in response to transmembrane voltage changes [42]–[45], although their maximal charge calculated from the transient current is at least an order of magnitude less than that of prestin [5]. The rapid electromechanical conversion manifested as somatic motility is, however, a unique property of prestin. The lack of motor function in non-mammalian orthologs, as we demonstrated in this study, indicates that the “voltage sensor” (to detect voltage change) and “actuator” (to generate length change and force) in the molecule may evolve independently and have different structural bases, although the two are believed to be fully coupled in mammalian prestin [28], [29], [46]–[48]. It is also likely that non-mammalian orthologs lack a unique structural element that couples the voltage-dependent charge movement to mechanical displacement. The recently identified 11–amino-acid motif that is unique to mammalian prestin may represent a structural adaptation that facilitates motor capability of mammalian prestin [33]. Nevertheless, we caution that we cannot rule out the possibility of small motility (less than 5 nm) in fPres and lPres that might be below the 5-nm resolution limit of our measurement system.

Based on differences in both amino acid sequences and the electrophysiological characteristics, a major episodic change(s) in prestin occurred with evolution of prestin in mammalian species, including the prototherians (i.e., platypus and echidna) and metatherians (e.g., opossum). The gain of motor capability is accompanied by diminishing transport function during evolution [9]. The acquisition of motor properties in mammalian prestin also coincided with the cytoarchitectural changes in the inner ear with the evolution of an organ of Corti from the basilar papilla of birds and reptiles in formation and the specialization of the lateral wall of outer hair cells in mammals [3], [12], [13]. Subsequent adaptive changes also appear to have occurred in prestin with the evolution of echolocation in various bat and aquatic mammalian species [38], [49]–[51]. We recently identified a segment of 11 amino acids in mammalian prestin that is remarkably conserved among mammalian species but highly variable among non-mammalian orthologs [36]. Substitution of this 11-amino-acid motif confers pendrin, a chloride/iodide transporter, and zebrafish and chicken prestins with motor-like function [33], [39]. This motif may represent the structural adaptation that enables motor function in mammalian prestin [39]. This motif is located within the SulPtp domain that encompasses the transporter machinery [1], [8] and represents an indel site [36]. To date, little if any changes in this motif is observed in over 40 eutherian species in the genomic sequence database (http://ensembl.org and http://www.ncbi.nlm.hih.gov/genome) that contain SLC26A5 genomic sequences (unpublished data). This site represents a major episodic shift within the sequence SLC26A5 of the SulPtp domain in the evolution of all the mammalian lineages. Both the prototherian platypus and metatherian opossum prestin exhibited NLC with the corresponding motor capability being more similar to gerbil than chicken prestin [9], [38]. The absence of this motif in the fPres and lPres provides additional evidence that the mammalian motif confers motor properties. The similarities of the fPres and lPres with the zebrafish and chicken prestins at the region of the mammalian motif suggest fPres and lPres retain anion transport capability.

Our previous studies have demonstrated that indels can contribute to the acquisition of motor-like properties in mammalian prestin [36]. There is a dichotomy between the therian/mammalian and non-mammal vertebrate sequences in four indel sites. There are four major segments containing insertions and deletions (indels) in the amino acid sequences that are distinct to gPres from lPres and fPres (Fig. 5). These indels are located with the following regions: one is associated with the mammalian motif, two sites are within the charged cluster domain and the fourth is in the carboxyl terminus. It is unlikely that the indel site within the carboxyl-end of the prestin peptide has any effect on prestin function, since truncation of these residues does not alter NLC and plasma membrane targeting [52]. The two sites within the charged cluster domain may influence the folding of the positively and negatively charged clusters of amino acids as well as the spatial orientation of the STAS domain within the cytoplasm and in its positional relationship with the SulPtp domain. In general, these differences are likely an indication of a significant transition from an electrogenic anion transporter to a unique molecular motor.
